# Target specific serologic analysis of COVID-19 convalescent plasma

**DOI:** 10.1371/journal.pone.0249938

**Published:** 2021-04-28

**Authors:** Sachie Ikegami, Robert C. Benirschke, Hossein Fakhrai-Rad, Mohammad H. Motamedi, Rick Hockett, Sean David, Hong Kee Lee, Jason Kang, Thomas J. Gniadek

**Affiliations:** 1 Department of Pathology and Laboratory Medicine, NorthShore University HealthSystem, Evanston, Illinois, United States of America; 2 Genalyte Inc., San Diego, California, United States of America; 3 Department of Family Medicine, Pritzker School of Medicine, University of Chicago, Chicago, Illinois, United States of America; Consiglio Nazionale delle Ricerche, ITALY

## Abstract

This study compared the performance of four serology assays for Coronavirus Disease 2019 (COVID-19) and investigated whether COVID-19 disease history correlates with assay performance. Samples were tested at Northshore using the Elecsys Anti-SARS-CoV-2 (Roche Diagnostics), Access SARS-CoV-2 IgG anti-RBD (Beckman Coulter), and LIAISON SARS-CoV-2 S1/S2 IgG (DiaSorin) as well as at Genalyte using Maverick Multi-Antigen Serology Panel. The study included one hundred clinical samples collected before December 2019 and ninety-seven samples collected from convalescent plasma donors originally diagnosed with COVID-19 by PCR. COVID-19 disease history was self-reported by the plasma donors. There was no difference in specificity between the assays tested. Clinical sensitivity of these four tests was 98% (Genalyte), 96% (Roche), 92% (DiaSorin), and 87% (Beckman). The only statistically significant differences in clinical sensitivity was between the Beckman assay and both Genalyte and Roche assays. Convalescent plasma donor characteristics and disease symptoms did not correlate with false negative results from the Beckman and DiaSorin assays. All four tests showed high specificity (100%) and varying sensitivities (89–98%). No correlations between disease history and serology results were observed. The Genalyte Multiplex assay showed as good or better sensitivity to three other previously validated assays with FDA Emergency Use Authorizations.

## Introduction

The Coronavirus Disease 2019 (COVID-19) pandemic created an urgent need for serology tests against a novel viral pathogen. Multiple companies rapidly developed assays to detect antibodies against the causative agent, SARS-CoV-2. These in vitro diagnostic tests were then submitted to the United States Food and Drug Administration (FDA) for Emergency Use Authorization (EUA), which permitted their marketing. Alternatively, in-house developed assays were validated as laboratory developed tests (LDTs).

Severe acute respiratory syndrome coronavirus 2 (SARS-CoV-2) is known to bind to the angiotensin-converting enzyme II (ACE2) receptor in order to gain entry to target cells. Similar to the first severe acute respiratory syndrome coronavirus (SARS-CoV), it appears that a region of the coronavirus spike protein known as the receptor binding domain (RBD) is primarily responsible for the SARS-CoV-2-ACE2 interaction. Interestingly, the spike protein is “primed” for ACE2 binding via an interaction with another human protein called TMPRSS2 [1]. Therefore, antibodies that bind regions of the spike protein other than the RBD may also inhibit viral infection. Finally, the nucleocapsid protein of SARS-CoV-2 has also been identified as highly immunogenic. Although the antiviral activity of anti-nucleocapsid antibodies are unclear, their presence may be sensitive and specific for prior exposure to SARS-CoV-2 [2].

SARS-CoV-2 are composed of 16 nonstructural proteins and 4 structural proteins; spike (S), envelope (E), membrane (M), and nucleocapsid (N). The *DiaSorin LIAISON® SARS-CoV-2* [3] is an indirect chemiluminescent immunoassay (CLIA) to detect IgG antibodies against the S1 and S2 spike proteins of SARS-CoV-2 virus. The *Beckman Coulter Access SARS-CoV-2* [4] is a two-step enzyme immunoassay to detect IgG antibodies specific for the receptor binding domain (RBD) of the S1 protein of SARS-CoV-2. The *Roche Elecsys® Anti-SARS-CoV-2* [5] is a chemiluminescent double-antigen sandwich immunoassay to detect antibodies that are able to bind nucleocapsid of SARS-CoV-2. All three assays have received Emergency Use Authorization (EUA) from the U.S. Food & Drug Administration (FDA) [6]. Unlike those assays, the Genalyte Maverick Diagnostic System simultaneously detects antibodies against multiple SARS-CoV-2 targets.

Primarily due to the novel nature of COVID-19, the correlation between antibody reactivity and immunity to relapse or re-infection is unknown [7]. Nevertheless, serology tests may be useful to identify which individuals have been exposed to COVID-19 in the past and identify who could donate COVID-19 convalescent plasma [8]. However, COVID-19 serology testing may be complicated by the highly variable clinical presentation of the disease [9]. It is possible that individuals with mild disease, certain demographics, or with certain symptoms may show variable serologic responses that affect assay sensitivity.

## Materials and methods

### Ethics statement

The NorthShore COVID-19 Convalescent Plasma Collection Study was approved by the NorthShore Institutional Review Board (EH20-170). Written informed consent was obtained from convalescent plasma donors. Control samples collected prior to December 2019 were residual anonymized specimens derived from clinical samples, for which Western IRB granted an exception to informed consent for research use (WIRB#20161322).

### Participant recruitment and inclusion/exclusion criteria

Convalescent plasma samples used in this study were collected from donors who had been recruited in April and May 2020. Recruitment efforts included messages sent to physicians within the NorthShore University HealthSystem, messages posted on internal websites, and listing on a national website for convalescent plasma collection centers. Participants were required to be greater than 18 years old, have recovered from COVID-19 greater than two weeks prior to consent, provide documentation of a positive SARS-CoV-2 PCR result confirming their diagnosis, and test negative by repeat nasopharyngeal SARS-CoV-2 PCR prior to sample collection. Potential donors were directed to call or email the NorthShore Blood Bank.

### Samples

One hundred frozen clinical samples collected prior to December 2019 by Genalyte were used as negative controls. These serum samples were collected in serum separator tubes, centrifuged for 15 minutes at 4,000 RPM, then stored in 1 ml aliquots at -80 C. All transported samples were shipped on dry ice. Samples from individuals who were confirmed to have COVID-19 were taken from convalescent plasma donors who volunteered for the NorthShore University HealthSystem COVID-19 Convalescent plasma collection study. NorthShore plasma donor samples were collected in serum separator tubes (BD vacutainer SST 367986), spun at 4,500 RPM for 7 minutes (EBA20 Hettich, Westphalia, Germany), and aliquots were frozen at -80 C.

### Transport and storage

Samples were collected from convalescent plasma donors in BD serum separator tubes (Becton, Dickinson and Company, Franklin Lakes, NJ). The tubes were spun, ~1ml aliquots were made, and the aliquots were frozen at -80°C. An aliquot from each donor was shipped overnight on dry ice to Genalyte for testing; another aliquot was thawed and tested on the three other assays at NorthShore.

### Serology testing

Four serology assays were used to analyze the specimens. Three of these are assays with current Emergency Use Authorizations (EUA) from the Food and Drug Administration (FDA) and adapted for use on automated high-throughput chemistry analyzers. Each of these assays targets a single antigen of the SARS-CoV-2 virus and testing was performed in a CLIA accredited laboratory at NorthShore University Health System (Evanston, IL). The final of these is a novel assay manufactured by Genalyte (San Diego, CA) which probes for antibodies against multiple SARS-CoV-2 antigens and other viral antigens. Specimens are then determined to be positive or negative for SARS-CoV-2 antibodies through the use of a multivariate machine learning model. This testing was performed in San Diego at Genalyte’s headquarters.

Aliquots tested at NorthShore were run on three automated assays: Elecsys Anti-SARS-CoV-2 (Roche Diagnostics), Access SARS-CoV-2 IgG (Beckman Coulter), and LIAISON SARS-CoV-2 S1/S2 IgG (DiaSorin). Each sample was tested on the same day using all three platforms. Each of these assays targets a different epitope of the SARS-CoV-2 virus and employs a different immunoassay format. The DiaSorin assay detects IgG antibodies against the S1 and S2 spike proteins of SARS-CoV-2 virus in an indirect chemiluminescent format. The Beckman assay detects IgG antibodies against the Receptor Binding Domain (RBD) of the S1 protein in a two-step immunoassay format. The Roche assay detects all antibody subtypes that are able to bind nucleocapsid viral antigens in a chemiluminescent sandwich immunoassay format. The Roche, Beckman, and DiaSorin assays are run on automated instruments with high specimen throughput capability, but relatively large physical footprint and startup costs. Conversely, the Genalyte assay is run on a non-automated device with relatively lower throughput, but also lower up-front acquisition costs and smaller physical footprint.

### Genalyte assay technology

The Genalyte assay employs multiplex detection technology based on silicon photonics that uses ring resonance to measure binding of macromolecules to sensors on a miniature silicon chip. The Maverick Diagnostic System detects changes in resonance wavelength as macromolecules, such as antibodies, bind to their respective antigens that are bound to the chip. Maverick™ SARS-CoV-2 Multi-Antigen Serology Panel (Genalyte Inc) is designed to detect IgG and IgM class of antibodies to five SARS-CoV-2 antigens within a multiplex format based on photonic ring resonance technology [10, 11]. A multi-antigen analysis algorithm analyzes individual qualitative results in order to generate an overall positive or negative result.

Genalyte’s Multi-Antigen Analysis Algorithm (MAAA) employs the well-known Random Forest Ensemble method with 3000 decision “trees”. Random forest is a classifier consisting of a collection of decision trees {*h*(***X***, Θ_*k*_)}, *k* = 1,…*n* where the {Θ_*k*_} are independent identically distributed random vectors and each tree casts a unit vote for the most popular class at input *X*. In particular, each new training set is drawn, with replacement, from the original training set. Then a tree is grown on the new training set using random feature selection. The generalization error of a forest of tree classifiers depends on the strength of the individual trees in the forest and the correlation between them. In the random forest model, the number of features to consider when looking for the best split at each node is set to $\left\lceil \sqrt{d}\right\rceil$ where d denotes the total number of features in the data. The Gini Impurity function is used as a measurement for the quality of splits [12].

### Donor demographics and history

Convalescent plasma donor COVID-19 disease history was obtained at the time of participant consent via a donor history screening form and blood donor screening questions (see S1 Fig). Free-text responses were converted into discrete data elements for the purpose of analysis. Whenever an abnormal finding was not reported by a donor, it was assumed to be absent / normal.

### Target-specific analysis

Genalyte assay data provided target-specific quantitative response broken down into IgG and IgM-based reactivity. The target-specific data from the 5 SARS-Cov2 targets was compared with the qualitative results of both the Beckman and DiaSorin assays to determine whether the results of the Genalyte targets correlated with the likelihood of the Beckman and DiaSorin assays being positive. P values were calculated with two-tailed Fisher’s exact test on a contingency table of true positives versus false negatives. The Haldane-Anscombe correction was used to calculate real-valued odds ratios for instances when a zero-valued denominator would have otherwise been present [13].

### Statistical analysis

Due to a lack of a priori knowledge of the expected range of serology test results for antibodies against a SARS-CoV-2, a power calculation was not performed prior to initiation of this study. The significance of pair-wise differences in sensitivity between the four assays tested was determined using a two-tailed Fischer’s exact test on a 2x2 table of true positives and false negatives. Target-specific analysis of the samples producing a false-negative result on the Beckman and DiaSorin assays was performed using the Fischer’s exact test on the qualitative results of both anti-spike protein assays along with the target-specific results of the Genalyte assay.

The Maverick TM SARS-CoV-2 Multi-Antigen Serology Panel v2 utilizes a multi-analyte analysisalgorithm (MAAA) to make the determination on patient samples of positive or negative orindeterminate for antibodies to the SARS-CoV-2 virus. The algorithm employed is a well-knownensemble method called Random Forests Classification. Random Forests contain a number ofdecision trees constructed from randomly chosen features that each make predictions on the data set, the aggregation of which gives the final result. These models are capable of fitting complex datasets and are resistant to overfitting.

Our implementation of the method uses 3000 such decision trees sampled randomly from training data and validated against test data. The model was also cross validated using five-fold cross validation. Three models were trained, and the combined IgG and IgM model proved to be the most robust, and is the model carried forward to call samples positive or negative or indeterminate for antibodies to SARS-CoV-2.

## Results

### Convalescent plasma donor characteristics

There was no statistically significant difference in donor gender, age, symptom duration, time from symptom resolution to sample collection, or difference in the presence or absence of any of the symptoms measured (see Table 1) between donors who tested positive by Beckman or DiaSorin and donors who tested negative with those assays (p > 0.05). Overall, the population of donors in this study reflected middle aged individuals who had symptoms but were not hospitalized for COVID-19.

**Table 1:** COVID-19 convalescent plasma donor information.

Donor Characteristics	Value
Total Number of Donors Tested	97
**Gender:**	
Male	54
Female	43
Age, years	49.5 (range 21–75, stdev 13.6)
COVID-19 Symptom Duration, days	14.8 +/- 9.9
Number of Donors without symptoms	1
**Reported symptoms of COVID-19 disease:**	
Maximum Temperature, F	100.4 +/- 1.7
Fatigue, %	53.6
Febrile, %	48.5
Cough, %	48.5
Loss of taste/smell, %	44.3
Headache, %	32.0
Sore throat, %	25.8
Chills, %	23.7
Shortness of breath, %	21.6
Diarrhea, %	21.6
Received treatment, %	17.5
Chest tightness, %	9.3
Skin rash, %	9.3
Night sweats, %	8.2
Hospitalized for COVID-19, %	4.1
**Symptom Resolution to Sample Collection, days**	43.5 (range 16–86, stdev 14.3)

### Clinical sensitivity and specificity

The total antibody assays (Roche and Genalyte) showed higher sensitivity compared with the IgG assays (Beckman and DiaSorin), see Table 2. The lowest sensitivity assay was the Beckman IgG (87%) and the highest sensitivity was with the Genalyte assay (98%). The only sensitivity differences that were statistically significant were between Beckman versus Roche (p = 0.0395) and Beckman versus Genalyte (p = 0.0054). All four assays demonstrated 100% specificity, without any false positive results among the 100 negative samples tested.

**Table 2:** Performance of COVID-19 serology assays.

	Total	True Positive	False Positive	True Negative	False Negative	Accuracy (%)	Sensitivity (%)	Specificity (%)
**Beckman IgG**	197	84	0	100	13	93	87	100
**DiaSorin IgG**	197	89	0	100	8	96	92	100
**Roche Total**	197	93	0	100	4	98	96	100
**Genalyte Total**	197	95	0	100	2	99	98	100

### Target-specific analysis

**Fig 1:**
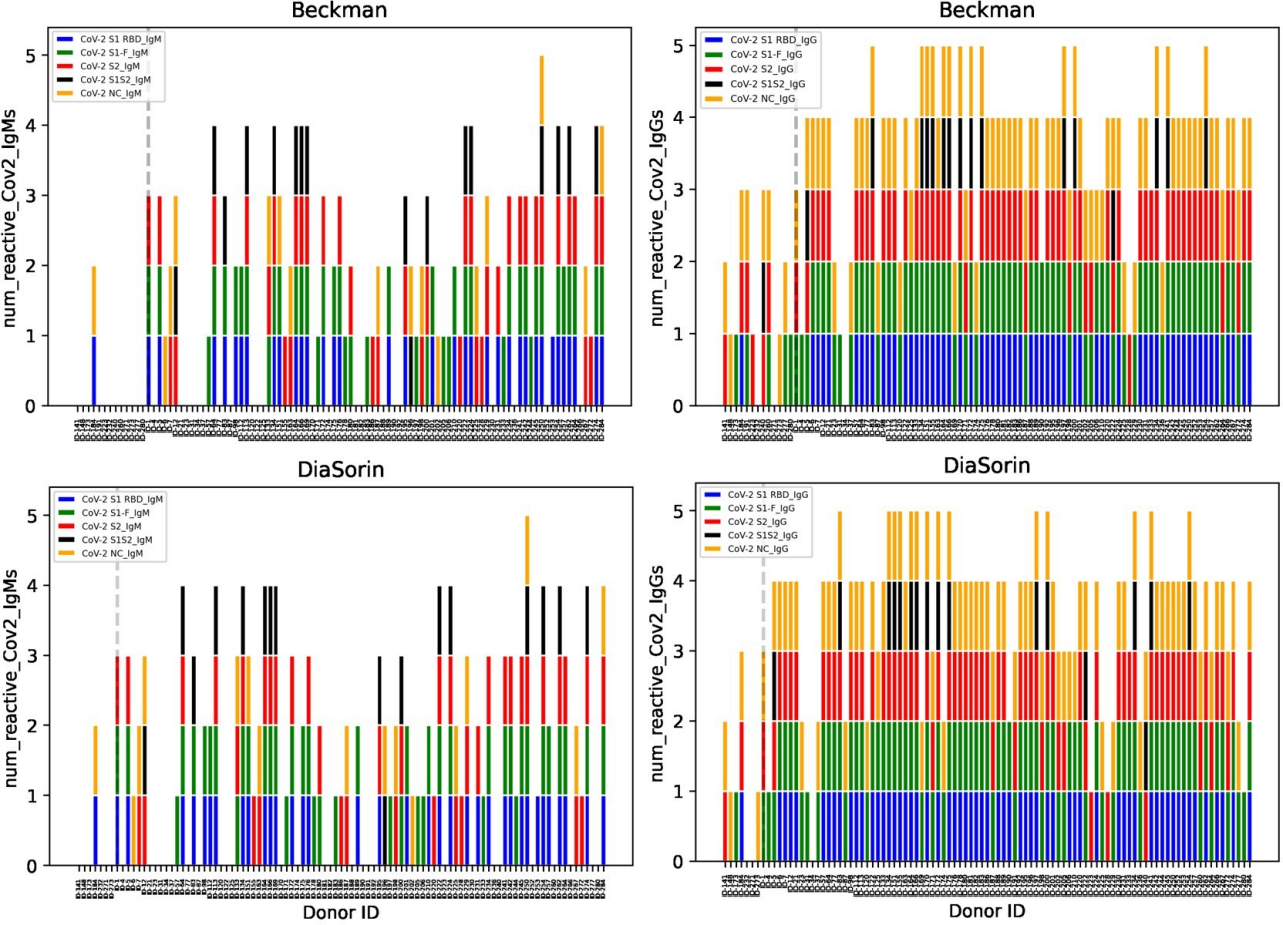
Comparison of Genalyte target-specific results with Beckman and DiaSorin results. The number of Genalyte assay reactive SARS-CoV-2 IgM (left) or IgG (right) specific targets for samples that were also tested on the Beckman (top row) and DiaSorin (bottom row) assays are shown for each sample tested. In each plot, false negative samples on the Beckman and DiaSorin assays are shown to the left of the dotted grey line.

Among the samples from patient’s with PCR-proven COVID-19, the Genalyte assay detected anti-S1-F IgG most often and anti-NC IgM the least often (see Table 3). Samples that resulted in false negative results on the DiaSorin and Beckman assays tended to have fewer positive targets on the Genalyte assay (Fig 1). Odds ratio calculation (Tables 4 and 5) showed that samples positive by Beckman and DiaSorin had a significantly increased likelihood of having positive anti-SARS-CoV-2 S1-F IgG (Beckman: p<4.0e-7, DiaSorin: p<1.5e-6), anti-SARS-CoV-2 RBD IgG (Beckman: p<1.5e-5, DiaSorin: p<0.01), anti-SARS-CoV-2 NC IgG (Beckman: p<0.006, DiaSorin: p<0.03) and anti-SARS-CoV-2 S2 IgG (Beckman: p<0.015, DiaSorin: p<0.01) on the Genalyte assay, compared with samples negative by Beckman and DiaSorin. Anti-SARS-CoV-2 S1-F IgM, anti-SARS-CoV-2 S2 IgM, and anti-SARS-CoV-2 S1 RBD IgM were positive more often in Beckman-positive samples compared with Beckman negative samples (p<4.5e-4, p<6.0e-4, and p<0.03 respectively), but showed no significant correlation with the DiaSorin assay result. Anti-SARS-CoV-2 S1S2 IgG and IgM as well as anti-SARS-CoV-2 NC IgM did not correlate with either Beckman or DiaSorin results.

**Table 3:** Genalyte assay target reactivity.

Analyte	Reactivity* (%)
SARS-CoV-2 S1-F IgG	89.7
SARS-CoV-2 NC IgG	88.7
SARS-CoV-2 S2 IgG	76.3
SARS-CoV-2 S1 RBD IgG	63.9
SARS-CoV-2 S1-F IgM	44.3
SARS-CoV-2 S2 IgM	41.2
SARS-CoV-2 S1 RBD IgM	37.1
SARS-CoV-2 S1S2 IgG	17.5
SARS-CoV-2 S1S2 IgM	17.5
SARS-CoV-2 NC IgM	16.5

The percentage of samples reactive for each SARS-CoV-2 target by the Genalyte assay is shown for the 97 PCR positive samples.

**Table 4:** Odds ratio of Genalyte target reactivity for true positive versus false negative samples by Beckman’s assay.

Genalyte Target	Estimated proportion of True Positive (SE)			
	Reactive	Non-reactive	P-Value	Odds Ratio [95% CI]
anti SARS–CoV– 2 S1 RBD_*IgG*_	0.984 (0.016)	0.657 (0.08)	1.14E-05	31.83 [CI: 3.91, 258.77]
anti SARS–CoV– 2 S1 F_*IgG*_	0.943 (0.025)	0.2 (0.126)	3.62E-07	65.6 [CI: 10.92, 394.23]
anti SARS–CoV– 2 S2_*IgG*_	0.919 (0.032)	0.696 (0.096)	1.17E-02	4.96 [CI: 1.47, 16.78]
anti SARS–CoV– 2 S1S2_*IgG*_	0.941 (0.057)	0.85 (0.04)	4.54E-01	2.82 [CI: 0.34, 23.32]
anti SARS–CoV– 2 NC_*IgG*_	0.907 (0.031)	0.545 (0.15)	5.83E-03	8.12 [CI: 2.02, 32.69]
anti SARS–CoV– 2 S1 RBD_*IgM*_	0.972 (0.027)	0.803 (0.051)	2.76E-02	8.57 [CI: 1.06, 69.0]
anti SARS–CoV– 2 S1 F_IgM_*	0.989 (0.016)	0.755 (0.058)	4.23E-04	28.3 [CI: 1.63, 491.51]
anti SARS–CoV2–2 S2_IgM_*	0.988 (0.017)	0.767 (0.055)	5.96E-04	24.57 [CI: 1.41, 426.79]
anti SARS–CoV– 2 S1S2_IgM_*	0.972 (0.039)	0.833 (0.041)	1.16E-01	7.0 [CI: 0.4, 123.62]
anti SARS–CoV– 2 NC_*IgM*_	0.938 (0.061)	0.852 (0.039)	6.88E-01	2.61 [CI: 0.31, 21.63]

Haldane–Anscombe correction has been implemented to calculate a real-valued odds ratio (*). SE stands for standard Error.

**Table 5:** Odds ratio of Genalyte target reactivity for true positive versus false negative samples by DiaSorin’s assay.

	Estimated proportion of True Positive (SE)			
Genalyte Target	Reactive	Non-reactive	P-Value	Odds Ratio [95% CI]
anti SARS–CoV– 2 S1 RBD_*IgG*_	0.984 (0.016)	0.829 (0.064)	8.36E-03	12.62 [CI: 1.45, 109.73]
anti SARS–CoV– 2 S1 F_*IgG*_	0.989 (0.011)	0.4 (0.155)	1.43E-06	129.0 [CI: 12.4, 1342.43]
anti SARS–CoV– 2 S2_*IgG*_	0.973 (0.019)	0.783 (0.086)	7.68E-03	10.0 [CI: 1.79, 55.8]
anti SARS–CoV– 2 S1S2_IgG_*	0.972 (0.039)	0.907 (0.032)	3.48E-01	3.57 [CI: 0.19, 65.55]
anti SARS–CoV– 2 NC_*IgG*_	0.953 (0.023)	0.727 (0.134)	3.00E-02	7.69 [CI: 1.46, 40.58]
anti SARS–CoV– 2 S1 RBD_*IgM*_	0.972 (0.027)	0.902 (0.038)	2.53E-01	3.82 [CI: 0.44, 33.08]
anti SARS–CoV– 2 S1 F_IgM_*	0.989 (0.016)	0.864 (0.046)	1.63E-02	13.74 [CI: 0.76, 247.71]
anti SARS–CoV2–2 S2_IgM_*	0.988 (0.017)	0.871 (0.044)	3.91E-02	12.03 [CI: 0.67, 216.99]
anti SARS–CoV– 2 S1S2_IgM_*	0.972 (0.039)	0.907 (0.032)	3.48E-01	3.57 [CI: 0.19, 65.55]
anti SARS–CoV– 2 NC_*IgM*_	0.938 (0.061)	0.926 (0.029)	8.70E-01	1.2 [CI: 0.13, 10.71]

Haldane–Anscombe correction has been implemented to calculate a real-valued odds ratio (*). SE stands for Standard Error.

## Discussion

When SARS-CoV-2 serology assays were initially developed, there was concern that false-positive rates in these assays would be high due to cross reactivity with antibodies formed against non-SARS-CoV-2 coronaviruses [14]. While the results presented here cannot exclude the possibility of false positive results when larger sample sizes are tested, it appears that multiple assay manufacturers have taken steps to minimize the chance of a false positive. Perhaps due to these efforts to prevent false positive results, these assays then could have substantial rate of false negative results [15].

The false negative results detected here were most common with the assay that uses the fewest number of SARS-CoV-2 epitopes as its target, in particular the RBD domain of the spike protein. Although this domain is clearly important for viral infectivity, it is likely that antibodies against other viral epitopes may also diminish viral infectivity. This could occur by inducing or preventing conformational changes in viral proteins that are necessary for fusion with the cell membrane as well as by promoting viral clearance through opsonization. However, it is important to note that binding antibody strength does not necessarily correlate with neutralizing antibody titer.

Finally, this study tested the hypothesis that false negative results on serology assays may correlate with certain patient characteristics (e.g. age, gender) or COVID-19 disease symptoms. Such a correlation was not found for false negative results from the two anti-spike assays. This study included insufficient numbers of false negative results from the other two assays tested to draw a meaningful conclusion.

Given the lack of COVID-19 PCR testing in the United States during the initial weeks of the SARS-CoV-2 pandemic, the high rate of very mild or asymptomatic infections, and the real possibility of false negative PCR assays, serology testing will likely remain a critical component of COVID-19 clinical diagnostics in addition to epidemiologic study. Although it remains unclear whether antibodies against certain SARS-CoV-2 epitopes can confer immunity to reinfection, it is apparent that the serologic response to COVID-19 is heterogeneous [16].

## Supporting information

S1 Fig (TIF)
