# Supplementary figures and images for: Target specific serologic analysis of COVID-19 convalescent plasma
Source: PLoS One. 2021 Apr 28;16(4):e0249938. doi: 10.1371/journal.pone.0249938 (PMC8081213; doi:10.1371/journal.pone.0249938)

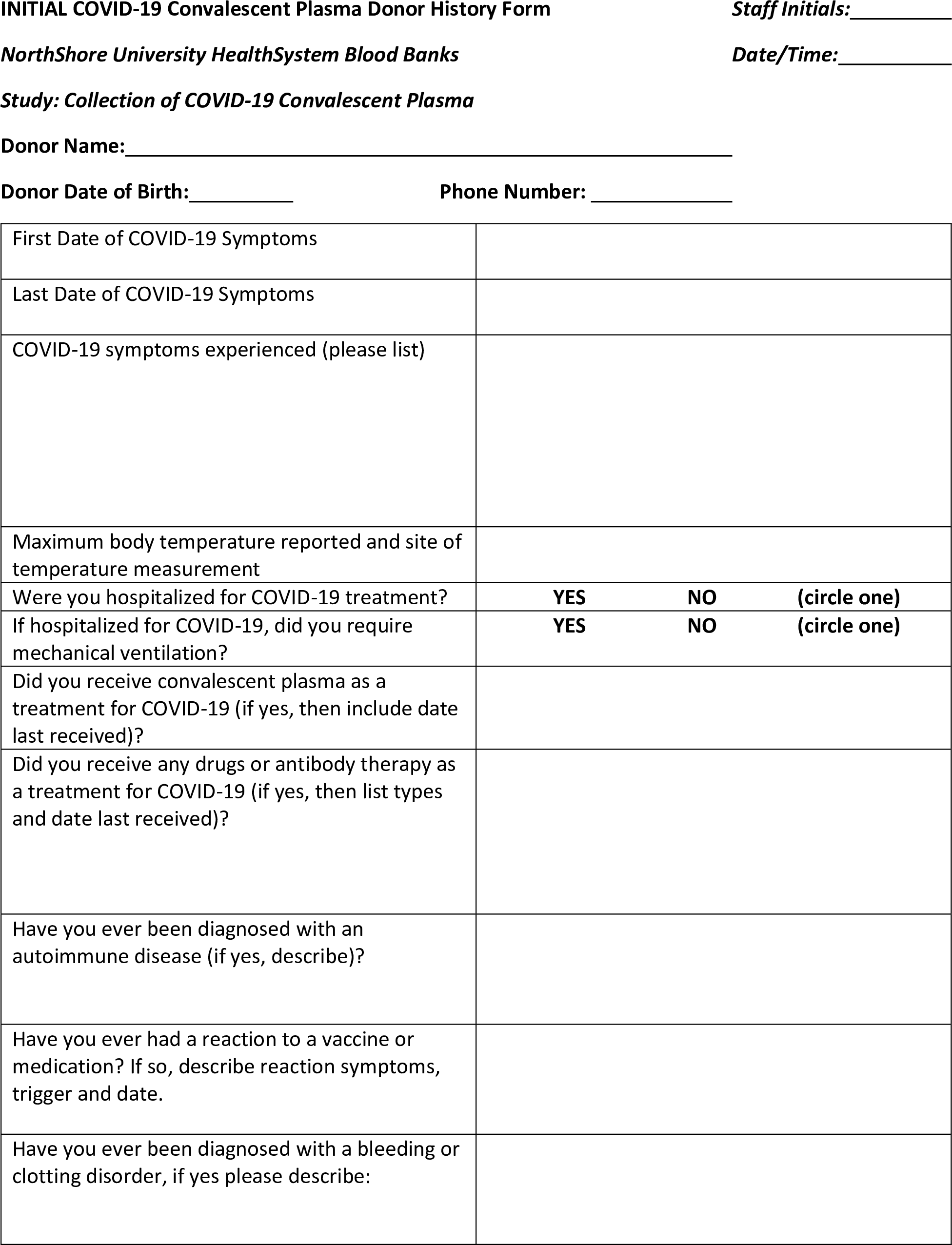

Supplement: S1 Fig — (TIF) [file pone.0249938.s001.tif]
